# Guyana’s paediatric training program: a global health partnership for medical education

**Published:** 2017-04-20

**Authors:** Lita Cameron, Julie C Johnstone, Arnelle Sparman, Leif D Nelin, Narendra C Singh, Andrea Hunter

**Affiliations:** 1 Department of Family Medicine, McMaster University, Ontario, Canada; 2 Department of Paediatrics, Hospital for Sick Children, University of Toronto, Ontario, Canada; 3 Georgetown Public Hospital Corporation, Georgetown, Guyana; 4 Department of Paediatrics, The Ohio State University, Ohio, US; 5 Guyana Help the Kids, Toronto, Ontario, Canada; 6 Department of Paediatrics, McMaster University, Ontario, Canada

## Abstract

Guyana is a low-middle income country on the northern coast of South America between Venezuela and Suriname. Guyana has relatively high child mortality and a notable gap in health care provision. As of 2011, there were no paediatricians in the public sector where approximately 90% of the population seek care. In response to this unmet need, Guyanese diaspora living in Canada, in partnership with Canadian paediatricians and the main teaching hospital, Georgetown Public Hospital Corporation (GPHC), developed a Master’s program in paediatrics. The postgraduate program was designed with adapted training objectives from the Royal College of Physicians and Surgeons of Canada and the American Board of Paediatrics. Innovative strategies to overcome the lack of qualified paediatric faculty in Guyana included web-conferencing and a volunteer North American paediatric faculty presence at GPHC with a goal of 1–2 weeks every month. By November 2016, 10 graduates will have passed through a rigorous program of assessment including a two-day final examination with an objective structured clinical examination (OSCE) component.

## Introduction and country context

The following is a brief narrative of a locally delivered, sustainable paediatric post-graduate program in Guyana that highlights Canada’s contribution to global medical education. This effort was led by Canada-based Guyanese diaspora and Canadian paediatricians in response to Guyana’s high child mortality rate compared to other Caribbean and South American countries.^1^

Guyana is former British Colony and member of the Caribbean Community (CARICOM). It is located between Surinam and Venezuela along the northern coast of South America, with a population of 799 600.^2^ The country is divided into ten regions, with an ethnically diverse community composed of East Indians (42.5%), those of African heritage (30.2%), and an Indigenous population of Amerindians (9.2%).^2^ The majority of the country’s inhabitants live in rural regions, with 20% in the capital city, Georgetown.^3^

Classified as a lower-middle-income country by the World Bank,^4^ Guyana is ranked as 118th of 186 countries by the Human Development Index (HDI).^5^ Guyana’s investment in healthcare utilizes 5.6% of the GDP, in combination with donor contributions.^6^ There is a publically funded health system with over 200 health centres throughout the country, ten district hospitals, five regional hospitals, and two national hospitals (including one psychiatric facility).^7^ The private sector includes six private hospitals, with five of these located in the capital.^7^

Guyana has a life expectancy of 66.3 years, and an under-five mortality rate of 37 per 1000 live births, ranking 65^th^ in the world by UNICEF.^1^ The infant mortality rate (40/1000 live births) and neonatal mortality rate (30/1000 live births), as well as the total estimated number of under-five deaths at 1000 per year, place Guyana amongst the lowest health indices for the region.^1^

## Medical education

In 1985, the University of Guyana (UG) School of Medicine was established, offering a six-year training program to high school graduates. Medical graduates complete a one-year internship to become a General Medical Officer (GMO). As of 2010, approximately 250 physicians have graduated from UG, but the majority had emigrated.^7^ Following internship, physicians work as GMOs in various departments, usually without formal specialist training. In response to a physician shortage, Cuba sponsored Guyanese medical students to train at the Latin American School of Medicine, Havana campus, and return to complete their final year in Guyana.^8^ It is estimated that as of 2013, 278 medical trainees have returned to Guyana, nearly doubling the number of physicians in the country.^7^ Currently, there are an estimated 0.214 physicians per 1000 population in the public sector^9^ (compared to 2.4 physicians per 1000 population in Canada).^10^

Guyana did not have formal post-graduate training programs available to medical graduates prior to 2006. In collaboration with the University of Guyana and the Georgetown Public Hospital Corporation (GPHC), a two-and-a-half-year Postgraduate Diploma in Surgery was established with the Canadian Association of General Surgeons.^11^ This was followed by postgraduate Diploma programs in Orthopedic Surgery and Anesthesia. In 2010, Guyana’s first three-year master’s degree residency program was initiated with a Master of Emergency Medicine (EM), led by Vanderbilt University.^12^ In 2012, Obstetrics and Gynaecology post-graduate training began in partnership with Case Western University. An Internal Medicine residency training began in 2013, initially with an international partner, and now with local leadership. In 2015, University of Ottawa’s Department of Family Medicine has begun to work with Guyanese partners to support primary care training.

## Paediatrics program

Prior to the establishment of the Master of Paediatrics program in 2011, there were three postgraduate-trained paediatricians in Guyana; these individuals were all internationally trained and worked in the private sector. The need for paediatricians is perhaps best illustrated by a comparison between the number of trained paediatricians and an estimated population of 397,000 children (under 18 years) in Guyana.^13^ The ratio of formally trained paediatricians to children in Guyana, based on this estimate, is 0.75 per 100,000 children compared to Canada, which is 7.5 per 100,000 children.^14^ Furthermore, the vast majority of Guyanese do not have access to the private sector, either due to location or lack of financial resources. The public sector paediatric wards were run by experienced GMOs and Registrars, who had exposure to intermittent paediatric training through some formal courses including: Neonatal Resuscitation Program (NRP), Paediatric Acute Life Support (PALS), and other general continuing medical education.^15^

Recognizing that locally trained medical staff are more likely to work locally and serve to minimize human resource gaps that occur when medical experts train outside their country^16^, a three-year Master of Paediatrics, accredited by the University of Guyana was established. This initiative was led by Canadian Guyanese diaspora and a charity, Guyana Help the Kids, in partnership with paediatricians from McMaster University and the University of Toronto. In the context of Guyana having one of the world’s highest emigration rates of highly skilled professionals,^17^ the lack of paediatric expertise in the country’s main teaching hospital presented a unique challenge with the development of a sustainable Guyanese led program. Key components around engagement, collaboration, and resources were identified in the development of the Master of Paediatrics and have been incorporated into the program to ensure local ownership and sustainability (Table 1).

The three-year structured Master of Paediatrics was accepted by the University of Guyana in early 2011, with the first cohort of postgraduates starting in November 2011. The program reflects the structure and major components of American and Canadian certifying organizations compiled into a simplified framework of 15 major subspecialty areas. The curriculum was designed by adapting objectives of training from the Royal College of Physicians and Surgeons of Canada, as well as the American Board of Paediatrics. These curriculum objectives were aligned with local resources and child health needs identified by local partners. Due to limited epidemiologic data of common paediatric illness in Guyana, extrapolation from similar middle-income countries within the Caribbean and elsewhere was necessitated. Content experts were consulted for further development of each subject area, and these continue to evolve with emerging analysis of inpatient data and in response to faculty and trainee experiences in the field.^18^–^20^

The residency program includes a series of one to three month rotations in paediatric inpatient wards, neonatal nursery, outpatient clinics and available subspecialty areas (see Table 2), as well as participation in case-based learning, journal clubs, academic half-day teaching sessions, case presentations, research/scholarly projects and development of evidence-based clinical protocols.

Initially, the program relied on part-time visiting faculty support with an in-country presence of up to two weeks per month. However, as local faculty have taken on leadership roles, there is more reliance on local resources. Canadian and American residents are also engaged through visiting electives in teaching and research, as well as liaison with local trainees to allow shared access to academic half-day sessions at McMaster University, via web-conferencing.

Assessment of trainees includes monthly written evaluations by local supervisors, ongoing brief clinical encounter feedback, written tests following each teaching block, and an annual written and OSCE exam comprised of relevant components from the “in training exam” from the shared Canadian pediatric residency programs in-training examinations. These exams are modified to the Guyanese context in terms of epidemiology and resources, and mapped to the curriculum.

The first cohort of post-graduate trainees included two experienced clinicians who have been longstanding leaders in the public hospital paediatric wards, who were expedited to graduate in 2014. Most trainees are GMOs working within the paediatric department prior to entering the program. Four of these individuals completed the program in 2015, and another four graduated in Fall 2016. This raised the number of postgraduate-trained paediatricians in the country to thirteen, and all ten of the graduates have remained engaged in the public sector. As the capacity for inpatient neonatal and paediatric care matures, the program has taken initial steps to progress towards a network of regional sites for inpatient care, as well as partnership with the emerging family medicine, neonatal nursing and midwifery programs towards providing improved primary and inpatient regional care for children throughout Guyana.

The training program has also intentionally fostered subspecialty areas of interests in most of the initial graduates, resulting in successful evolving programs in paediatric echocardiography, oncology treatment, allergy and asthma clinics, and neonatal care. These graduates all have competency and plan to continue clinical practice in general paediatrics, with the added skills and confidence that sub-specialization affords, as many will be located in regional hospitals with ongoing links to the tertiary hospital and continued involvement in the residency training program.

Multiple clinical protocols have been developed and implemented, as well as over 15 works of original research presented at the annual Guyana Medical Scientific Conference, and a number presented outside Guyana, as well.

## Next steps

One next step is the formal evaluation of this training program to date. Efforts are underway to identify elements of the evaluative process, including paediatric data, where available (GPHC in-patient, outpatient clinic and Emergency Department data), resident feedback, and qualitative interviews with new graduates and program partners.

In addition to program evaluation, infrastructure needs to be strengthened to increase data collection and analysis throughout the country, particularly in rural and district hospitals. Data on child health in Guyana is sparse, providing challenges for monitoring progress. Initial analysis of inpatient data for 2012 at the GPHC paediatric inpatient ward identified pneumonia, acute lower respiratory tract infection, gastroenteritis, asthma, and ingestion among the top five reasons for admission to hospital (Table 3). Research is currently being undertaken to analyze trends of inpatient admissions including length of stay, diagnosis and mortality. This will inform the evaluation of the program and identify further educational needs.

Retention of health professionals will also be a key evaluative component of this postgraduate training program. A local program director is now established, with ongoing support from international partners, and all 10 graduates of the program are engaged in positions within the public sector.

As the program matures, the focus can shift from training the core group of paediatricians to support the large tertiary care centre, to further facilitating regional and more rural/remote care for the significant proportion of Guyanese children who live substantial distances from the primary training site in the capital. Senior trainees have already engaged with regional hospitals as a component of their training program, and are encouraged to continue to work to build regional capacity in primary and secondary care of children with local health professionals.

## Discussion

The Master of paediatrics residency program is emerging as a sustainable training program for developing and supporting local resources. The ten graduates have all continued working in the public sector, and remain engaged and involved in the teaching and mentoring of residents within the program. This has resulted in a decreased need for external visiting paediatric faculty. More importantly, it allows for the continued development of a locally led Guyanese paediatric program. Other examples of paediatric training programs in low-resource settings include Laos, Cambodia, Eritrea and Kenya – where locally based two- to three-year training programs have led to a high percentage of graduates continuing to work in country.^21^

Guyana has a significant challenge in retention of highly skilled workers. Thus, the Ministry of Public Health and the partners in this program will need to turn their attention to workforce needs for paediatrics in the public sector. The government will need to commit to methods for retention of paediatricians, including measures such as fair pay, reasonable working hours, etc. This commitment is a vital investment for the long-term sustainability of paediatrics as a specialty in Guyana and for continued improvement in the health of all children in Guyana, as this program is able to sustain presence in regional centres, and continue local capacity development.

The graduates of the program have also taken on important roles at GPHC in paediatric sub-specialties. Each of the four paediatricians in the last graduating class have partnered with subspecialists at various institutions in Canada and the United States (Paediatric Cardiology – University of Calgary; Respiratory medicine – University of British Columbia; Neonatology – The Ohio State University; and Paediatric Oncology – The University of Buffalo). For the first time in Guyana, these sub-specialty services can be offered in the public sector. These same graduates have taken on the medical director roles for the NICU, paediatric oncology, paediatric cardiology, and ambulatory care. As these roles mature through positive outcomes and development of other disciplines necessary to treat more complex conditions, the paediatric program in Guyana will need to certify and maintain practice standards for these sub-specialties.

Improved paediatric training and a commensurate increase in resources for medicines and medical infrastructure will fill a large gap in health care in Guyana, which will lead to an improvement in child health and a decrease in child mortality. An initial retrospective analysis of inpatient neonatal mortality at GPHC comparing 2010/11 and 2011/12 showed a substantial decrease in mortality (13.8% vs. 7.7%,) as a likely result of a new NICU, training of nursing staff, and the initiation of the paediatric training program.^18^

### Summary

We described a novel approach to developing a postgraduate training program in paediatrics in a lower-middle-income country that initially lacked qualified paediatricians in the main teaching hospital. Several academic departments in Canada and the United States partnered with the only tertiary teaching hospital in Guyana, the Ministry of Health, and the University of Guyana to develop this innovative program. This partnership provides an example of global health in medical education that can contribute to work-force training and retention.

## Figures and Tables

**Table 1:** Key components for a viable and sustainable training program

Canadian volunteers with a connection to Guyana, either through the pre-existing postgraduate training programs or personal connections with the Guyanese-Canadian diaspora, estimated to number at least 20,000 in the Greater Toronto Area. Within the country, strong leadership from the health sector, specifically the Ministry of Public Health and the University of Guyana. Due to the work led by other Canadian initiatives, UG established the Institute for Health Science Education at the GPHC to facilitate the courses and post-graduate training. Existing local physicians, particularly two GMOs leading paediatric care in the public sector who, upon formal graduation offered essential support for the education and training to interested GMOs as well as ongoing clinical supervision. Development of relevant curriculum, access to external examinations resources, and support for research including clinical protocol development through associated academic affiliations to McMaster University and the University of Toronto. Availability of some funding for key clinical equipment, courses and visitors.

**Table 2:** Rotation schedule for three-year postgraduate training program

**Year 1**	3 months General Paediatric Ward 3 months NICU 2 months Ambulatory Paediatrics 1 month Accident and Emergency 1 month Community Paediatrics 1 month Infectious Diseases
**Year 2**	2 months General Paediatric Ward 2 months Ambulatory Paediatrics 1 month NICU 1 month ICU 1 month Surgery Clinics 1 month Community Paediatrics 1 month Obstetrics and Gynecology 1 month Development and Adolescent Medicine 1 month elective
**Year 3**	1 month teaching block 1 month A&E 1 month research 3 months elective 2 months Junior Consultant 1 months Ambulatory Paediatrics 2 months Community Paediatrics

**Table 3:** Admission diagnosis for GPHC Paediatric Ward inpatients^22^

Admission Diagnosis	Number of Patients
Pneumonia	150
Acute Lower Respiratory Tract Infection	140
Gastroenteritis	139
Asthma	109
Ingestions	107
Sickle Cell	67
Malaria	56

n=1380
